# Everybody's Page

**Published:** 1889-06-29

**Authors:** 


### Everybody's Page.

Some doctors look upon hay fever as a hereditary disease.

An anonymous donor has expressed his intention of presenting the People's Palace Library with a thousand volumes. May his example be widely followed.

It should never be forgotten in scarlet fever that however mild the case, there is always a liability to dropsy in the later stages of the disease.

There is hope of protection for the lizard, who has lately been martyred that his skin may be made into pocket-books and purses. The Government of Java have issued an edict forbidding the further slaughter of lizards in that island, as they are useful in destroying a number of noxious insects.

Is the daily bath as necessary to our welfare as we think? Dr. Fridthof Nausen, who looks the picture of health and manly strength, confessed that in crossing Greenland he did not wash for over two months; and even adds that if he had indulged in the luxury of a tub, it would have rendered him much more liable to painful blistering by the Arctic sun.

The ingenious author of "English as She is Spoke" has been beaten by Mr. K. S. Okamoto, of Tokio, who has published a work on the usages of Japan. The book is illustrated, and many of the illustrations are confusing, but not nearly so much so as the English explanations of them. For example, the "hair-tool of women" is a little vague. Is the tool in question a comb, a brush, or a hairpin? Many pages are taken up with pictures of the games of children, carefully divided into "amusements of male," "amusements of female," and "amusements of male and female." Among the last, Mr. Okamoto includes "to look fiercely each other." No doubt both boys and girls do look so occasionally; but where does the fun come in?

The go-a-head spirit of America is apt occasionally to make it forget the courtesies, one might say the decencies, of life. Recently, a well-known and popular officer of the United States Army committed suicide in a fit of temporary insanity. The next day there appeared in several New York papers the advertisement of a firm of quack medicine vendors, headed "The Death of Captain Witthans," and stating that if the unfortunate gentleman had only taken a bottle of their particular remedy he would never have suffered from the madness that brought about his end. English advertisers have used an English officer's name to popularise somebody's pills, and the escape of the "Calliope" to tell the public of somebody else's asbestos, but hitherto we have spared the dead.

Mr. Edward Berdoe told a good story at the meeting of the Anti-Vivisection Society last week. A woman came to the London Hospital complaining of what was called a skin disease. The clever but sarcastic physician to whom she applied for treatment, saw at once that the "skin disease" was of a peculiar but not very uncommon nature. "I should advise you, madam," he said, with great courtesy, "to fill a bath with water of a temperature of 90deg., and to immerse yourself in it; then take some yellow soap, rub it vigorously on a piece of flannel, and apply the flannel with considerable friction to all parts of your body." "But doctor," said the patient, "that sounds like telling me to go home and wash myself." "I confess, madam," was the reply, "that it is open to that objection." She never came back.

In some recent cases of typhoid, which have been traced to impure milk, it seems that the primary source of infection was the stagnant water of the ponds from which the cows drank.

It is better to use coarse flannel than fine for fomentations. There is more air in the interstices of the former, and for this reason it will keep warm longer, air being a bad conductor of heat.

Mrs. Booth, who is suffering from cancer, has greatly benefited by a course of electric treatment. Medical electricity may therefore count on having the influence of the Salvation Army—no mean thing—to support it.

An American lady doctor, who is herself an enthusiastic cyclist, strongly recommends cycling as a form of exercise specially suitable for women. She has been practising medicine for seventeen years, and in the course of her practice became convinced that more vigorous movement of the lower limbs than walking gives was necessary to the proper development of her patients. This, she thinks, is given by cycling.

National characteristics are hard to eradicate. There is still some truth in the description given of the Gauls by Julius Cæsar, nineteen centuries ago: "The Gauls have a love of revolution; they allow themselves to be led by false reports into acts which they afterwards regret, and into decisions on the most important events. They are depressed by reverses; they are as ready to go to war without cause as they are weak and powerless in the hour of defeat."

It is possible to give a scientific aspect to the old fatalistic idea that no man dies till his time has come, and no man can evade his doom. Disease germs are almost everywhere around us, we inhale them every day, yet some of us escape unhurt, while others succumb. The reason of this is that each constitution has a predisposition to certain diseases and an insusceptibility to others. These predispositions form a sort of soil where one disease will develop while another will decay. One man may have a predisposition to typhoid as surely as another may have a predisposition to consumption, and even under similar circumstances neither will contract the other's disease.

There is no courage like the courage of a conscientious spiritualist. Sir John Franklin went to the Arctic regions, and never returned; his wife remembered that he had mentioned an alternative route by which he would return if his expedition failed, and, acting on this information, Captain (now Sir Leopold) M'Clintock sailed northward in the "Fox," and found relics that proved the fate of the previous explorers. These are the generally-accepted facts of the subject; but, in face of it, a reverend believer in spiritualism, Mr. J. H. Skewes, has the courage to tell the world that the discovery of Sir John Franklin's remains was due to a revelation made by a spirit named "Weesey" to a child of a certain Captain Coppin, of Londonderry. If this be so, why has "Weesy" waited two-and-thirty years before claiming the credit of her discovery, meanwhile letting her laurels rest on the head of Sir Leopold M'Clintock? The only way in which she can prove her claims now is to do something of the sort again. We should be obliged to "Weesy" if she will give us some accurate information as to the present position of Emin Pasha.

